# Effect of a Multi-Faceted Training Program on Falls in Senior Pickleball Players

**DOI:** 10.3390/healthcare13182298

**Published:** 2025-09-13

**Authors:** Betsy Myers, June Hanks

**Affiliations:** Department of Physical Therapy, College of Health, Education and Professional Studies, University of Tennessee at Chattanooga, Chattanooga, TN 37403, USA; june-hanks@utc.edu

**Keywords:** pickleball, injury, senior athlete, fall risk

## Abstract

Background/Objectives: Over half of older adults who play pickleball report falling during play, yet little is known regarding the ability of training programs to reduce the incidence of falls or fear of falling (FOF). The purpose of this study was to determine the effect of a multi-faceted training program for senior pickleball players who reported falling while playing pickleball or self-limiting play due to FOF. Methods: This cohort study included 12 participants in the training group (TG) and 17 in the control group (CG) who were at least 55 years of age, regularly played pickleball, and reported falling while playing or limiting play due to FOF. Hip abduction strength, change in direction (COD), and agility were assessed. Participants completed a survey regarding fall history and FOF. The TG participated in a 10-week program focused on strength, balance, COD, and agility. Results: At baseline, there were no between-group differences except that the CG participated in walking for exercise more often than the TG. The strength analysis showed no significant main effects of time or group, but there was a significant Group × Time interaction, F(1, 25) = 14.86, *p* < 0.001, η^2^ = 0.240. Simple effects analysis showed that the TG demonstrated a strong trend toward improvement (9.9 Nm increase, +5%, *p* = 0.091) while the CG significantly declined (18.4 Nm decrease, −12%, *p* < 0.001). The COD analysis revealed no significant main effects of time or group, but there was a significant Group × Time interaction, F(1, 26) = 8.21, *p* < 0.001, η^2^ = 0.373. Simple effects analysis showed that the TG significantly improved their COD time (0.7 s faster, *p* = 0.045) while the CG showed no significant change (0.25 s slower, *p* = 0.168). The TG had a significant decrease in FOF, z = −2.427, *p* = 0.015, r = 0.701. The agility analysis revealed no significant effects for time, group, or Group × Time interaction, F(1, 26) = 0.89, *p* = 0.354, with both groups showing minimal non-significant changes. At the end of training, the TG was playing 50% more pickleball than the CG, z = −2.192, *p* = 0.028. At the 5-month follow-up, 25% of the TG reported falling during play compared to 44% of the CG. Conclusions: A multi-faceted training program can effectively reduce FOF and improve physical performance in senior pickleball players.

## 1. Introduction

Pickleball has been the fastest-growing sport in the United States from 2021 to 2025 and is now a billion-dollar industry [1]. Pickleball provides moderate to vigorous intensity exercise [2] and has been shown to improve blood pressure control and lipid profiles [3]. Pickleball burns more calories than walking for the same amount of time despite using fewer steps [4], while also promoting social interaction [5]. The increase in popularity of the sport may partly be due to the lower overall body stress when compared to running or team sports, such as soccer and basketball, and a learning curve for competitive play that is less steep than similar sports, such as tennis or padel. Former tennis players report transitioning to pickleball due to curiosity about the sport, the social benefits of pickleball, and reduced body stress [6]. With roughly 15% of the United States population now engaging in the sport [1], the average age of pickleball players lowered from 55 years of age in 2016 [7] to 35 years of age in 2024 [1].

Despite the reduction in average player age, there has been an exponential increase in the number of pickleball-related injuries [8] and an estimated annual healthcare cost of USD 377 million [9]. Between 2020 and 2022, there was a significant increase in emergency department visits and hospitalizations from pickleball-related injuries, primarily among players aged 65 and older [10]. When examining both emergency department data [11] and single-center data [12], older pickleball players were more likely to be injured and more likely to sustain a fracture. Interestingly, while 82% of those injured had a previous background in sports [6], falls were the most common reason for emergency department visits [13]. In one study, 42% of players reported a pickleball-related fall, with 30% reporting falling more than once [14].

Falls have been reported by 16% to 38% of community-dwelling older adults [15], and up to half of these individuals reported a fall-related injury [16]. Over a third of community-dwelling older adults who fall will develop a fear of falling (FOF). Not only does FOF increase the risk of falls [17], it may also reduce function [15,18] and restrict participation in a wide range of activities, including attending outings in unfamiliar places, walking for physical activity, and meeting basic exercise guidelines for maintaining and improving overall health and wellbeing. Fear of falling is associated with a sedentary lifestyle [19] and reduced quality of life [20]. Importantly, FOF occurs equally in community-dwelling older adults who have fallen and those who have not fallen [18,20,21].

In addition to reducing fall risk and building confidence, exercise can reduce FOF [22], reduce depression [22], and is known to improve function and quality of life [23]. Hip abductor weakness [24], trunk weakness [25,26], and power deficits (slower lower extremity force production) [27] may increase the risk of falls. Multi-dimensional strength and balance training programs improve strength and mobility in community-dwelling older adults [28], reduce fall risk [28], and reduce FOF [29]. Balance training that involves dual tasking appears to be more effective in reducing falls than traditional balance exercises [30]. Change in direction (COD) and agility-based exercises are also recommended components of general fall reduction programs [31]. Functional training has been shown to be efficacious in reducing falls and FOF in community-dwelling older adults [32]. While studies have shown that regular participation in sports reduces the risk of falls [33,34], these studies did not differentiate between sport-related and non-sport-related falls. Additionally, the authors of this study were unable to identify the literature on the effect of training programs for older adults that target the reduction in falls that occur during sport participation.

Unfortunately, despite the significant number of pickleball-related falls and injuries, to date, there is no evidence to support methods to prevent falls or reduce FOF in senior pickleball players. Current recommendations for pickleball injury prevention and fall prevention remain largely anecdotal or extrapolated from general injury prevention principles. For example, experts recommend pickleball players wear proper footwear to reduce the risk of injury [3,35]. Additionally, a retrospective study suggests that limiting play sessions to under two hours and not playing on consecutive days can reduce pickleball-related injuries [36]. More broadly, factors associated with general sports-related injuries include an overall lack of preparedness for physical activity, limited warm-up prior to participation, and deficits in strength, COD abilities, and agility (reacting to a stimulus) [37]. Sport-specific injury prevention programs, such as the FIFA 11+ for youth football (American soccer), are known to reduce injuries by 39% [38] while also improving sport performance [39]. Pickleball training programs to reduce falls and FOF should extrapolate information gleaned from community-dwelling older adults while considering the specifics of pickleball play. Pickleball requires transfer of power from the lower extremities through the trunk to the upper extremity paddle hand, rapid changes in direction, multi-planar lunging, agility when reacting to an opponent’s shot, along with adequate balance to maintain body position while visually tracking the ball, and increasing the capacity to get to the ball. Training programs should address the leading reported reasons for pickleball-related falls: lunging and moving backward [14]. The hip abductors and hip extensors [40] are critical for pelvic control [41] during lunging, while the quadriceps [42] are key to decelerating and reversing lunge direction. Functional exercises such as squats and step-ups elicit significant activation of not only the quadriceps but also most hip musculature [41]. Footwork drills, such as line stepovers and moving from a jog into a ready position (wide stable base with knees bent) to strike the ball [43], may assist with injury and fall prevention. Therefore, a well-rounded training program for fall prevention and improving confidence should be multi-faceted and include lower extremity and trunk strength training, dual-task balance activities, COD and power exercises, and sport-related agility (reactive) drills. The purpose of this study was to determine the effect of a multi-faceted training program on senior pickleball players who reported falling while playing pickleball or self-limiting play due to FOF. The research hypothesis was that compared to a control group (CG), the training group (TG) would demonstrate improvements in strength, COD, and agility measures and report a reduction in FOF.

## 2. Materials and Methods

### 2.1. Study Design

Cohort study. Training sessions were held on Thursdays from 10:45 am to 11:45 am at the same location. Scheduling was based on facility and researcher availability. This strict schedule prevented randomization of participants into groups because many potential TG participants had other conflicting events, such as caring for grandchildren, volunteer work, or paying jobs during training times.

### 2.2. Participants

Recreational pickleball players were recruited from a southern city in the United States with a population of about 200,000 people via flyers, email, social media, and word of mouth. To be included in the study, participants were required to be at least 55 years of age, have reported that, on average, they played pickleball at least one hour one time per week without restriction (e.g., not limiting play due to injury or illness), and had either a history of falling during play or self-limiting play (i.e., “giving up on a ball” by not attempting to return a lob over their head) due to concern for injury or falling. Additionally, participants needed to read and understand English. To be eligible for the TG, participants also needed to be consistently available to attend most weekly training sessions. Individuals with progressive neurological conditions, such as Parkinson’s disease, were excluded from the study. Similarly, any individual judged to be unsafe with forward/backward/lateral movements by the primary researcher (a physical therapist) was also excluded from the study. An a priori power analysis with G*Power (version 3.1) using a 2 × 2 ANOVA for repeated measures within–between interaction, alpha of 5%, and 80% power found that 34 total participants would be required to detect a medium effect size of 0.5. Therefore, to allow for the possibility of participant dropout, a group size of 20 participants for both the TG and the CG was chosen.

### 2.3. Procedures

**Figure 1:**
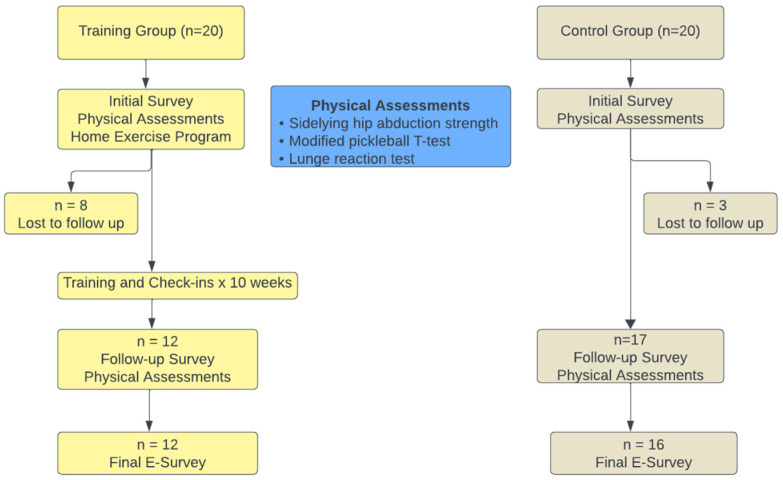
Study Format.

The study was approved by the Institutional Review Board of The University of Tennessee at Chattanooga. Written informed consent was obtained for all participants involved in the study. Individuals were placed in the TG if they wanted to participate in training and their weekly schedules permitted participation in at least 70% of the training sessions. Individuals who did not want to participate in weekly training or who had scheduling conflicts were placed in the CG. Once the maximum of 20 participants was reached in the TG, additional participants were asked to join the CG until the maximum of 20 participants was met. Figure 1 demonstrates the overall format of the study.

All participants completed an initial survey and physical assessments in person, with a follow-up approximately 3 months later. To obtain information regarding FOF, the survey included questions regarding their history of pickleball play questions adapted from the Perceived Control Over Falling Scale [44] and the Survey of Activities and Fear of Falling in the Elderly [45]. The nine questions regarding FOF were rated on a 3-, 4-, or 5-point Likert scale. Scores were summed for a total score (0–29), with lower scores indicating less FOF and greater confidence.

The initial and follow-up surveys were identical except that the TG was asked two additional questions to track their performance of their dynamic warm-up prior to playing pickleball and frequency of home exercise program (HEP) completion. To improve recall, the TG completed a weekly check-in survey to report changes in health status, number of times they played pickleball between sessions, performance of a warm-up prior to play, any pickleball-related falls, and physical activities outside of pickleball. To track changes over time, all participants were emailed a link to a final e-survey approximately five months later.

**Figure 2:**
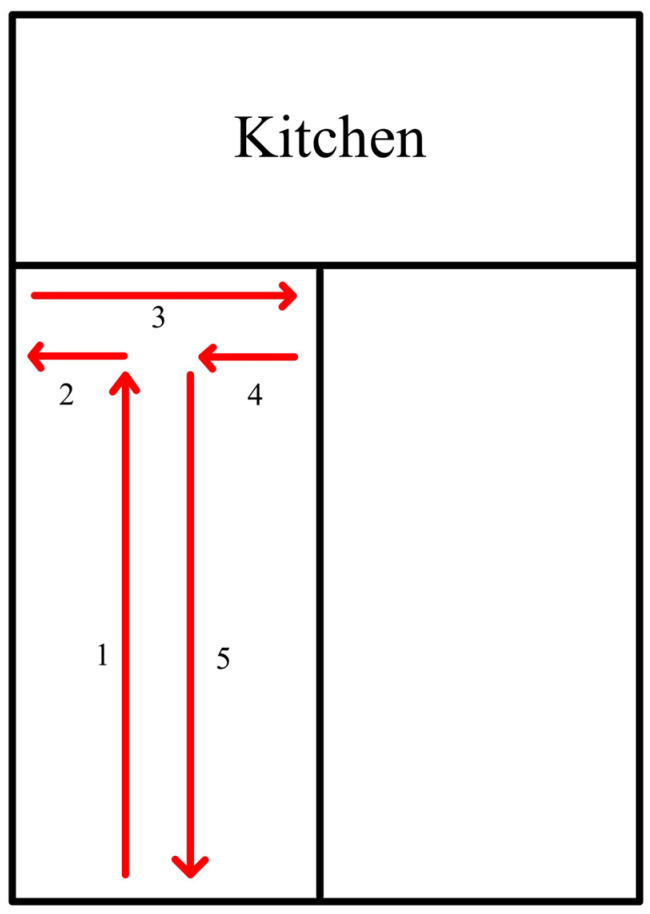
Modified pickleball *T*-test. The participant runs forward from the center of the left side of the pickleball court to the kitchen line (1), then shuffles sideways to the left to the sideline (2); then shuffles sideways to the right to the opposite sideline (3); then shuffles right to the center (4); and then turns to run forward to the endline (5).

**Figure 3:**
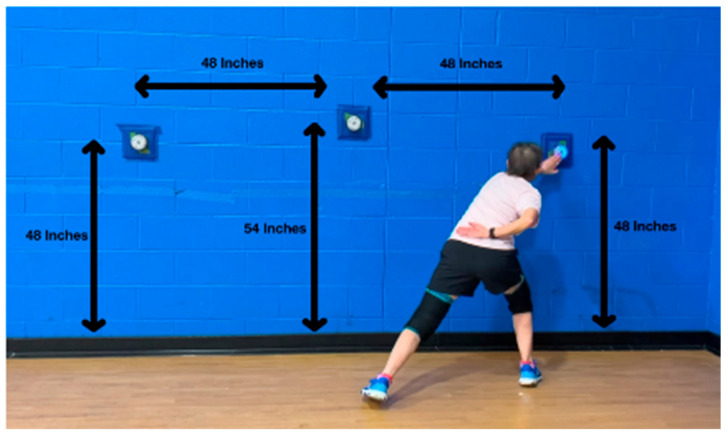
Lunge reaction test performed by a left-hand dominant pickleball player.

For the TG, training sessions were performed at the same time of day and on the same day of the week. For the CG, each participant’s initial and follow-up assessments were performed at the same time of day (either mid-morning or early evening). Participants performed their initial and follow-up physical assessments after a 5 min dynamic warm-up, including jogging, side shuffling, and lunging, followed by any individual stretching/warm-up they desired. Each assessment included one practice test followed by two recorded trials. The best trial was used for analysis. Each assessment was performed by one of two evaluators after achieving consistency in practice. Left and right hip abduction strength was measured with the participant lying on their side on a yoga-style mat with the top hip abducted to the horizontal. The assessor resisted the participant’s ability to hold the leg abducted horizontally using a handheld dynamometer placed just proximal to the lateral malleolus. The force was recorded to the nearest 0.1 kg. Leg length was measured only on the right leg from the greater trochanter to the lateral malleolus and recorded to the nearest cm. Hip strength was assessed as hip abduction torque (Nm), calculated using the formula: hip abduction force (kg) × leg length (m) × 9.81. Left and right hip abduction scores were totaled to create an overall strength measurement with higher values indicating greater strength. Change in direction was assessed using the modified pickleball *T*-test (Figure 2), a shortened Agility *T*-test that mimics movements within a typical doubles pickleball game. Time was recorded to the nearest 1/100th of a second using a stopwatch, with lower values indicating greater speed. Agility was tested using the lunge reaction test (Figure 3). Three BlazePods (BlazePod, Miami, FL, USA) were attached to a wall, with the participant starting in front of the center BlazePod. The left or right pod would randomly light up and be turned off by the participant lunging to tap the surface of the pod. Once the side pod was tapped out, the center pod would light up, and the participant would lunge to tap it out. This sequence (random left/right BlazePod followed by the center home BlazePod) continued for 30 s. The number of hits was recorded with greater values indicating better agility.

**Figure 4:**
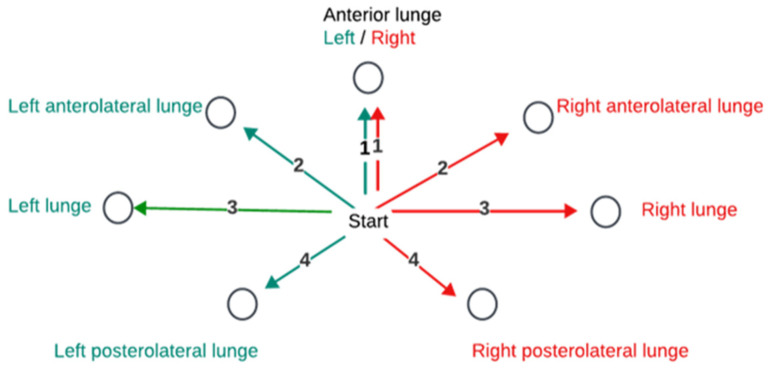
Lunge matrix. Left (green) and right (red) lunges are made in four directions, anterior (1), anterolateral (2), lateral (3), and posterolateral (4).

On the day of the initial assessment, the TG was instructed in and performed a targeted, sport-specific, progressive HEP that included bridging, side stepping with a resistance band, lunge matrix (Figure 4), plank, and a dual-task single-leg balance activity. Written copies, including three-step progressions for each exercise, were provided, and the TG was encouraged to perform the HEP 2–3 times per week. Training consisted of 10 weekly sessions of approximately one hour in length. Each session began with a standardized 5–10 min full-body dynamic warm-up that included movements similar to those required during pickleball play, such as squatting and lunging. Written copies of the warm-up were provided, and the TG was encouraged to perform this warm-up before playing pickleball outside of the training sessions. After the warm-up, the TG performed progressive movement competency drills that included fast feet, power, and balance exercises (Table 1) for approximately 5 min, followed by approximately 20 min of COD and agility activities (Table 2). Last, the TG performed approximately 20 min of progressive strength training (Table 3) moving through 5 stations beginning with 3–5 rounds of an exercise for 20 s on/20 s off per station. Each station had a researcher instructing participants in exercise technique, providing cues for form as needed, and encouraging progression if the current exercise intensity required less than moderate effort. Strength training exercises were progressed by increasing hold time and/or increasing resistance (e.g., add/increase weight, elevated to floor plank, wall sit at 45° to 90° of knee flexion, bilateral to unilateral bridge). Over the course of the 10 weeks of training, the time spent on agility drills was gradually increased from approximately 5 min to 15 min with a corresponding decrease in time devoted to performing COD drills.

### 2.4. Statistical Analysis

Between-group differences in demographics and physical activities were assessed using a *t*-test for scale data. However, due to the non-normal distribution identified using the Shapiro–Wilk test, activity level was analyzed using the nonparametric Mann–Whitney U test. The Mann–Whitney U test was also used to analyze between-group differences on the survey. Three separate 2 × 2 mixed model ANOVA tests were conducted to examine the effect of training on scale measures of strength, COD, and agility. Group (training versus control) served as the between-subjects factor and time (pre- versus post-intervention) as the within-subjects factor for each analysis. A chi-square test was used for categorical data (e.g., falls versus no falls). Significance level was set at 0.05. Effect sizes (η^2^ and r) were calculated for significant results. For this study, the strength of the effect size for r was defined as follows: <0.20 = small effect, 0.50 = medium effect, and 0.80 = large effect [46]. The strength of the effect size for η^2^ was defined as follows: <0.01 = small effect, 0.06 = medium effect, and 0.14 = large effect [47].

## 3. Results

### 3.1. Baseline

Twenty-nine participants (12 TG, 17 CG) completed both initial and follow-up testing. Eight individuals in the TG were lost to follow-up: two individuals attended only 1 training session, and six attended less than 30% of sessions. Reasons for discontinuing training included family illness (2), personal illness (2), and multi-joint arthritis flare-up (1). Two individuals discontinued the study due to a desire to play pickleball rather than train. These individuals reported joining the study solely because doing so was the only way for them to continue to play at their regular day, time, and location. Once they found a new location to play, following their previous schedule, they no longer participated in the training. Fourteen of the seventeen (82.4%) individuals in the CG voiced a desire to participate in the TG but were unable to do so either due to the training schedule or location (11) or the TG was full (3). At baseline, there were no differences between groups regarding demographics, survey responses, or physical activities, with the exceptions that the CG participated in more walking activities (z = −2.857, *p* = 0.004) and more balance exercises (z = −2.264, *p* = 0.024) than the TG (Table 4).

### 3.2. Changes in Performance Measures

Participants in the TG attended an average of 86% of the training sessions (range 70–100%). The TG reported performing their HEP an average of 2.6 times per week (SD = 1.12, range 0.8–4.5) and warm-up 0.6 times per week (SD = 0.42, range 0–1.0). Results of performance measures by group and time are presented in Table 5.

Table 6 provides a summary of key results. The TG had significant improvements in FOF with a medium to large effect size. For the 2 × 2 mixed model ANOVA tests, there were no outliers, as assessed by examination of studentized residuals for values greater than ± 3. All scores were normally distributed as assessed by the Normal Q-Q Plot. There was homogeneity of covariances, as assessed by Box’s test of equality of covariance matrices, and homogeneity of variances as assessed by Levene’s test of homogeneity of variances. The strength analysis revealed no significant main effect of time or group. There was a significant Group × Time interaction with a large effect size. The groups showed virtually identical baseline performance. The significant interaction reflected opposing change patterns with the TG improving by 9.9 Nm (+5%) while the CG declined by 18.4 Nm (−12%), resulting in a 28 Nm differential training effect. Simple effects analysis showed that the TG demonstrated a strong trend toward improvement at the end of the study (*p* = 0.091) while the CG significantly declined (*p* < 0.001). The COD analysis revealed no significant main effect of time or group. There was a significant Group x Time interaction with a large effect size. Simple effects analysis showed that the TG significantly (0.7 s faster, *p* = 0.045), while the CG showed no significant change (0.3 s slowed, *p* = 0.168). The agility analysis revealed no significant effects for Time, Group, or Group × Time interaction. While there was a similar trend in agility scores, with improvement in the TG from pre- to post-intervention and decline in the CG, these changes for both groups were small and non-significant.

At the end of the intervention, the only difference between groups in activity level was that the TG was playing significantly more pickleball than the CG, z = −2.192, *p* = 0.028 (mean 3.6 ± 1.67 for TG, mean 2.4 ± 1.37 for CG). At the 5-month follow-up, while not significantly different [Χ^2^(1) = 1.050, *p* = 0.306], 25% of the TG reported falling while playing pickleball compared to 44% of the CG, with one person from the CG lost to this final follow-up.

## 4. Discussion

This study provides the first evidence supporting a multi-faceted training program for reducing falls and FOF while simultaneously improving physical performance in senior pickleball players. The TG demonstrated significant improvements in FOF with medium to large effect sizes and a clinically relevant trend of reduced pickleball-related falls. Additionally, the TG experienced significant gains in COD speed and maintenance of strength, while the CG declined significantly in these measures. These findings have important implications for addressing the growing number of pickleball-related falls and injuries in older adults.

Building confidence is crucial to reducing FOF [20]. The 33% improvement in FOF scores observed in the TG represents a clinically meaningful change, supporting that the multi-faceted program enhanced participants’ perceived control over falling. The perception of control over falling matters greatly as low confidence can lead to panic responses that increase the risk of falling and can restrict activity [45]. The increase in the TG’s reported frequency of pickleball participation during the follow-up period suggests that the reduced FOF and associated improved confidence during play may have ultimately increased sport participation.

The slight gains in strength by the TG, coupled with the significant declines in the CG, are particularly noteworthy for fall prevention and improved sport performance. Prior research has shown that reduced hip abductor strength may increase fall risk in older women [48]. Additionally, hip abductor strength is critical for pelvic control during lunging, running, and side-stepping [49], which are movements common in pickleball. Likewise, the TG’s improvement in COD speed represents not only a substantial enhancement of sport-specific performance but may also have contributed to the reduction in FOF. The relationship between COD performance and fall risk may be complex, as greater COD time has been correlated with greater FOF in community-dwelling older adults [50], suggesting that older pickleball players may proactively self-limit their speed during testing to ensure safety due to heightened FOF. More specifically, pickleball players reporting a pickleball-related fall had significantly greater COD times than those who did not [14]. The progressive training program may have helped build confidence, allowing TG participants to move at faster speeds that may have more closely replicated pickleball play, and ultimately reduce fall risk. There also appears to be an interaction between lower-body strength and COD ability, as in both tennis [51] and netball [52], strength gains are associated with improved COD abilities.

While COD is a recommended component of fall-reduction programs and is considered an important determinant of athletic performance [53], COD represents a closed, pre-planned skill, while agility is an open, reactive skill that includes both COD speed and decision-making ability in response to a stimulus. Because COD tests do not appear to accurately represent agility [54,55], the inclusion of sport-specific agility testing is considered essential [56]. The lunge reaction test consisted of pickleball-specific movements (alternating lateral lunging in response to a visual stimulus). While participant height may have influenced the difficulty of the test (length of lunge), this should not affect changes in performance over time. However, the uniplanar nature of the test and task time (30 s) may have failed to adequately mimic the sport of pickleball. The absence of change on the lunge reaction test, despite improvements in other domains, suggests that the test may lack the sensitivity required to detect subtle performance changes. It is also possible that cognitive abilities involved in agility, such as stimulus recognition, require more time or greater training exposure than changes in strength or COD. For example, in young soccer players, training frequency (performing at least two training sessions per week) was more important in gaining agility than the training program duration [57]. More specific to the senior pickleball population, a successful strength and agility program for fall prevention in community-dwelling older women was performed twice weekly for 25 weeks [58]. For safety purposes, the training program in this study progressed from simple COD activities to more challenging reactive drills weekly over a 10-week period. Additionally, the prescribed HEP did not include an agility component. Therefore, while the changes in agility scores followed the same trend as FOF, strength, and COD scores (improvement in the TG and decline in the CG), the training dose for agility may have been too low in this study to create a significant change.

The high session attendance (86%) demonstrates the feasibility of implementing such programs in recreational pickleball settings. The social support provided through the group training sessions may have enhanced home exercise program adherence [59]. There was a high initial dropout rate from the TG primarily due to personal or family illness (50% of dropouts). This may be due to the increased vulnerability of older individuals to illness or due to the training occurring during flu season. The low adoption of a pickleball-specific warm-up suggests that additional education and behavioral change strategies may be needed, particularly given that performing a dynamic warm-up is such a strongly recommended injury prevention strategy [60].

There are a few limitations to this study. The lack of random assignment to groups introduces the potential for selection bias. While the groups did not differ in age, sex, or play characteristics, and the majority of the CG verbalized a desire to participate in the training, the groups may have differed in motivation or other unmeasured characteristics, limiting the generalizability of the study. While the 5-month follow-up data demonstrating lower fall rates in the TG suggest benefits from the training were sustained, this short follow-up period limits the ability to understand the long-term effects of the training program. Last, while significant differences were found in strength, COD, and confidence, the study may have been slightly underpowered to detect smaller changes in agility given a total of 29 versus the preferred 34 participants in the a priori power calculation. Future research should include randomized control trials with larger sample sizes and a longer follow-up period. Training may require increased time or frequency to safely and substantially challenge agility, given the age of the participants and comparison with sport-specific programs such as FIFA 11+.

## 5. Conclusions

This study provides preliminary evidence that a multi-faceted training program can effectively reduce FOF and improve physical performance in senior pickleball players. Training program participants increased strength, improved COD speed, gained confidence, reported fewer falls, and had higher rates of sport participation at follow-up. Given the recent increase in pickleball-related injuries in seniors, implementing evidence-based fall prevention programs in recreational settings represents a promising strategy for reducing injury burden while meeting recommended physical activity guidelines in an enjoyable, social setting.

## Figures and Tables

**Table 1:** Fast feet, power, and balance exercises.

	Description	Progressions
Fast Feet	Jog in place, then attain a ready position on command Line step overs	None Lateral and front-to-back line step overs progressed via increase in speed
Power	Lunge matrix Skater progression	Lunge matrix * Skater progression * Push to ready position (Stand in ready position, push laterally with outside foot, land in ready position) Half skater (Stand on one leg, push off laterally, land on opposite foot, pause, repeat) Full skater (Stand on one leg, push off laterally, land on opposite leg, immediately push back to start position, repeat)
Balance	Partner (or wall) ball toss and catch	Note, if a participant was unable to stand on one leg while performing a secondary task, the drill was performed with a wider base of support, either in tandem stance (slightly easier) or with feet together(significantly easier) Partner ball toss Partner/wall dink rally: Cooperative/gentle hit of the pickleball to bounce in front of partner (or wall) who then returns in the same manner Partner/wall volley rally: Cooperative/gentle hit of the pickleball in the air to partner (or wall) who then returns in same manner

* Progression via increase in range, then speed.

**Table 2:** Change in direction and agility activities.

	Description	Progressions
Change in Direction	Planned change in direction drills performed over half-court-width (~10 feet).	Single lateral shuffle in one direction Single lateral shuffle to lunge Continuous lateral shuffle left and right Figure-of-8 lateral shuffle with 3 cones ¾ box shuffle (lateral shuffle left, forward jog, lateral shuffle right) Shuffle to fast jog (lateral shuffle left or right to quick jog forward) Figure-of-8 vertical drill with 3 cones Drop step to jog
Agility	Reactive drills where participants responded to a stimulus (BlazePod, researcher, or ball) to change directions	Reactive lateral lunge Reactive 3-direction (anterior, anteriolateral, lateral) lunge Reactive 3-direction (anterior, anteriolateral, lateral) jog/shuffle Reactive half-court drills. From the middle of the baseline with pickleball paddle, participant ran forward and responded to a stimulus to complete one of the following activities. Shuffle left or right Return a dink from the left or right Turn in response to a lob over their left/right shoulder and jog to catch the ball after it bounces Turn in response to a lob over their left/right shoulder and hit the ball to return it to the other side of the court

**Table 3:** Strength training exercises.

Strength training exercises
Goblet squats (wide-based squat with weight held in hands at chest) or wall sit Chop/lift (diagonal pull-down/pull-up patterns) with resistance band Step-ups or bridge (from hook lying supine position, raise buttocks up off support surface) Core circuit of front plank and prone total body extension (superman) Resisted side-step with band.

**Table 4:** Baseline demographics and physical activities.

	Training Group	Control Group
Age (years) #	67.1 ± 5.2 (range 57–74)	65.2 ± 8.9 (range 57–84)
Sex		
Female	8	12
Male	4	5
Years playing #	2.1 ± 1.3	3.7 ± 3.9
Times per week playing pickleball #	3.61 ± 1.67	3.83 ± 1.6
Skill level †		
≤2.0	0	0
2.5	0	1
3.0	7	7
3.5	5	8
4.0	0	1
>4.0	0	0
Falls (number)	1.8 ± 1.8	1.6 ± 1.5
Walking ‡	0 (0–2)	3 (1.5–6) *
Jogging ‡	0 (0–0)	0 (0–0)
Cycling ‡	0 (0–0)	0 (0–0)
Swimming ‡	0 (0–0)	0 (0–0)
Strength training ‡	0 (0–3.75)	1 (0–2.5)
Racket sports other than pickleball ‡	0 (0–0)	0 (0–0.5)
Balance exercises ‡	0 (0–0)	1 (0–2) *

# Mean ± SD; † frequency; ‡ median (inner quartile range); * control group greater than training group.

**Table 5:** Performance measures by group and time.

Measure	Group	Intervention Time 1 (mean ± SD)	Intervention Time 2 (mean ± SD)	Change	Effect
Survey score	Training	9.2 ± 4.5	6.2 ± 3.4	−3 ‡	Improved
	Control	6.6 ± 3.8	7.6 ± 2.7	+1 ‡	Declined
Strength (Nm)	Training	177.9 ± 58.8	187.8 ± 57.3	+9.9	Improved
	Control	178.2 ± 38.9	159.8 ± 37.1	−18.4 *	Declined
	Total #	178.0 ± 47.0	171.2 ± 47.5		
Change in Direction (seconds)	Training	9.2 ± 1.9	8.5 ± 1.9	−0.7 *	Improved
	Control	7.7 ± 1.8	8.1 ± 1.7	+0.4	Declined
	Total #	8.4 ± 1.9	8.3 ± 1.8		
Agility (Hits)	Training	15.1 ± 1.6	15.5 ± 1.7	+0.4	Improved
	Control	16.1 ± 1.9	15.8 ± 2.1	−0.3	Declined
	Total #	15.4 ± 1.7	15.6 ± 1.9		

# Total = average of all participants; ‡ Significant between-group differences (*p* < 0.015); * Significant within-group change from Time 1 to Time 2 based on simple effects analysis (*p* < 0.05).

**Table 6:** Statistical summary of key results.

Measure	Effect	Statistic	*p*-Value	Effect Size
Survey score		z = −2.427	*p* = 0.015 *	r = 0.701
Strength	Time	F (1, 25) = 2.02	*p* = 0.261	
	Group	F (1, 25) = 0.59	*p* = 0.450	
	Group × Time	F (1, 25) = 14.86	*p* < 0.001 *	η^2^ = 0.240
Change of Direction	Time	F (1, 26) = 2.02	*p* = 0.167	
	Group	F (1, 26) = 1.83	*p* = 0.188	
	Group × Time	F (1, 26) = 8.21	*p* < 0.001 *	η^2^ = 0.373
Agility	Time	F (1, 26) = 0.26	*p* = 0.615	
	Group	F (1, 26) = 0.68	*p* = 0.419	
	Group × Time	F (1, 26) = 0.89	*p* = 0.354	

* = Significant difference.

## Data Availability

The original contributions presented in this study are included in the article. Further inquiries can be directed to the corresponding author.
